# Frontiers of Additively Manufactured Metallic Materials

**DOI:** 10.3390/ma11091566

**Published:** 2018-08-30

**Authors:** Amir A. Zadpoor

**Affiliations:** Additive Manufacturing Laboratory, Faculty of Mechanical, Maritime, and Materials Engineering, Delft University of Technology (TU Delft), Mekelweg 2, 2628CD Delft, The Netherlands; a.a.zadpoor@tudelft.nl; Tel.: +31-15-278-1051

**Keywords:** additive manufacturing, 3D printing, metals and metallic alloys, digital manufacturing, multi-scale manufacturing

## Abstract

Additive manufacturing (AM) (=3D printing) has emerged during the last few years as a powerful technological platform for fabrication of functional parts with unique complex geometries and superior functionalities that are next to impossible to achieve using conventional manufacturing techniques. Due to their importance in industrial applications and the maturity of the applicable AM techniques, metallic materials are at the forefront of the developments in AM. In this editorial, which has been written as a preamble to the special issue “Perspectives on Additively Manufactured Metallic Materials”, I will highlight some of the frontiers of research on AM of metallic materials to help readers better understand the cutting edge of research in this area. Some of these topics are addressed in the articles appearing in this special issue, while others constitute worthy avenues for future research.

## 1. Introduction

Additive manufacturing (AM) (=3D printing) is one of the most important developments in manufacturing since the turn of the century. Although the underlying technology has been under development for several decades, it is only during the last decade that the technology has matured to the level that is required for reliable fabrication of functional parts. The layer-by-layer manufacturing not only means that complex geometries could be achieved with no additional costs but also enables the realisation of geometries for which no feasible fabrication technique existed before. Moreover, the lead time is much shorter in AM as compared to many conventional techniques, while batch size makes little to no difference in terms of the production costs. A combination of all these advantages makes AM an extraordinarily promising manufacturing platform for a host of industrial applications. Metals and metallic alloys are the most commonly used types of materials in a wide range of industries. Moreover, research into AM of metallic materials started in the 90’s and has been continuing for more than two decades. As a result, the technology required for AM of metallic materials has matured and reached the level that enables direct fabrication of functional parts. That is one of the reasons why metal AM is currently receiving so much attention from both researchers in academia and actual industrial users.

Given the relatively high manufacturing costs, the first applications of AM have been in high added value industries such as biomedical (see e.g., [1]) and aerospace (see e.g., [2,3,4]) industries. Continuous research and development are, however, ongoing to decrease the costs associated with AM, thereby expanding the range of possible applications. Moreover, the mechanical properties of AM parts are not always as good as those achieved by conventional manufacturing techniques. In order to tackle both above-mentioned challenges, researchers are developing and improving the AM processes, the materials used in AM, and the post-AM processes that could be used for the improvement of the microstructure and mechanical properties of AM materials as well as for inducing new functionalities. In addition to experimental approaches, computational models could play an important role in designing and optimizing all these approaches. Furthermore, the complexity-for-free and batch-size-indifference features of AM have paved the way for the emergence of a whole new range of possible functionalities. Advanced design-for-AM techniques are required to take full advantage of such possibilities, and are currently being intensively researched. In what follows, I will briefly review the frontiers of research in the above-mentioned areas to highlight the most important topics of current interest and to set the stage for the papers appearing in this special issue.

## 2. AM Processes

The most commonly used metal AM processes, namely selective laser melting and electron beam melting being, are classified under the general category of powder bed fusion technologies. These techniques work based on layer-by-layer melting of selected areas within a powder bed. Direct energy deposition techniques are also used for processing of metals and metallic alloys, particularly when near net shape parts are desired, when the part being fabricated does not fit the build volume of the powder bed fusion machines, or when AM is used for the repair of already existing parts. Finally, some studies try to interface metal printing with other AM technologies such as those based on material extrusion to create hybrid materials [5].

Metal AM is successful only if the laser processing parameters are properly selected so as to simultaneously optimize three objective functions: fidelity of the built parts (parts conforming to CAD design), density of the parent material (≈100%), and the mechanical properties of the built material. The usual approach for selecting the processing parameters is trial and error. This approach is time-consuming, expensive, and inaccurate, particularly for geometrically complex parts. Moreover, the obtained parameters are often not transferable from one machine or lab to another. This presents a major challenge for every new geometrically complex part that needs to be manufactured using AM, and is a barrier to widespread application of cost-effective AM. First-time-right AM is an ideal approach to metal AM where the built part is fabricated without any need for trial and error. There is currently no first-time-right AM technology available that guarantees direct success of the AM process regardless of the geometrical complexity or material type. The success of any such technological platform is likely to be dependent on developments in three core underlying technologies including process monitoring techniques that provide a rich set of data for controlling and predicting the AM process, process simulation models that are capable of using the data provided by the process monitoring systems and predict the best processing parameters, and control systems enabling on-the-fly adjustment of processing parameters. Although all three types of technologies have received increasing attention during the recent years, the level of maturity, integration, and precision required for first-time-right AM has not yet been realised. Enhancing the capabilities of these core underlying technologies as well as integrating them into a functional and reliable first-time-right AM platform constitutes one of the most impactful directions of future research and one that is being pursued by several research labs worldwide.

Two main limitations set the AM of metallic materials apart from that of polymeric materials. First, there are few methods currently available for multi-material AM of metals. Second, the minimum feature sizes that could be achieved with metallic material is usually much larger for metals as compared to polymers. Development of processes and machines that allow for multi-material AM of metals with resolutions <50 µm should be a priority for future research. In particular, support materials that could be easily removed would enable fabrication of parts with clearances (e.g., joints) and small-angle geometrical features that are not possible to realise with the vast majority of currently available machines. Increasing the production rate of metal AM techniques through addition of more energy sources (e.g., laser beams) or re-design of build platforms are important areas that could benefit from further research too.

Finally, hybrid manufacturing equipment that combines AM techniques with subtractive manufacturing techniques in one single apparatus could increase the production speed, (surface) quality of the fabricated parts, and geometrical fidelity. The design of hybrid manufacturing systems including not only the equipment but also optimized control/planning algorithms are the other topics of current interest that could facilitate the adoption of AM and hybrid manufacturing techniques by various industrial sectors.

## 3. Materials for AM

AM techniques have been successful in fabrication of (functional) parts from a wide range of metallic materials including (but not limited to) titanium [6,7,8,9] and its light-weight high-strength alloys (e.g., Ti-6Al-4V [10,11,12,13]), Ti-Nb alloys [14], (stainless) steel [15,16,17,18], CoCr/CoCrMo alloys [19,20,21,22], shape memory alloys based on NiTi [23,24,25,26,27], high performance superalloys (e.g., Inconel [28,29,30,31]), Mg and its alloys [32,33], Zn [34], and Al alloys [35,36,37,38]. Obtaining parts that possess properties similar to those achieved by conventional manufacturing processes is one of the challenges of metal AM. What is perhaps less obvious at the first glance is that the best materials for metal AM are not necessarily the ones originally developed for conventional manufacturing processes. The characteristics of metal AM processes call for the development of new alloys that work the best with AM. Indeed, development of new materials tailored to the specific requirements of AM may be a more fruitful avenue of research than applying AM to the materials developed for other manufacturing process and trying to adjust the manufacturing parameters and apply post-AM processes to achieve the types of microstructure and mechanical properties that are comparable with those realised by the processes for which the materials had been developed in the first place.

A number of high profile studies into development of materials with exceptionally favourable properties have taken the pure materials science approach suggested here. For example, nanoparticle nucleants have been used in one study [39] to control the solidification process and enable AM of Al alloys that cannot be easily processed with AM techniques. Addition of other agents that influence the solidification process, in situ alloying [40], or pre-alloyed powders with novel chemical compositions could be all used for introducing new metallic materials for AM and to control their microstructure and mechanical properties. These areas of research have not so far received attention and require further research to clarify what the limits of achievable microstructures, mechanical properties, and physical properties are. The effects of the characteristics of the powder material [41] and build direction [42,43] on the AM process and the properties of the resulting materials need to be thoroughly investigated as well.

## 4. Post-AM Treatments

Even if the desired microstructure and mechanical properties are not achieved by adjusting the AM process itself (e.g., modifying the processing parameters [44]), there are possibilities to improve them after the AM process using post-AM treatments. The other application of post-AM treatments is to reduce manufacturing irregularities or to induce surface-related functionalities that could enhance the performance of AM metallic materials. As far as the first aim is concerned, heat treatments at elevated temperatures have been used for relieving the residual stresses and improving the microstructural features of AM materials [11,45,46]. The exact (local) heat treatments to achieve both goals simultaneously are not currently known for all processes, parameters, and geometries, and, thus, require further research. Moreover, computational models (see Section 5) may be needed to understand the spatial distribution of residual stresses and to design the (local) heat treatments such that the undesired stresses could be optimally relieved without having overly severe consequences for the mechanical properties of the AM material. Combining heat treatments with high pressures in processes such as hot isostatic pressing (HIP) [47,48,49] is another promising approach to not only achieve the above-mentioned goals in terms but also close the pores that may exist in the AM part. Once more, the best design of these post-AM processes and their applicability to different materials are not yet well understood. As a consequence, HIP treatments are not always successful in improving the mechanical behaviour of AM metals [50,51]. Further research is therefore needed to clarify what the best post-treatments are for every type of material and geometrical design.

Another approach for improving the mechanical properties of AM metals particularly their fatigue performance is the application of surface treatment techniques. Unmolten powders and the irregularities caused by the AM process could lead to the formation of a large number of potential crack initiation sites. For parts with simple geometries, the manufacturing irregularities could be decreased using such techniques as machining and polishing. For more complex parts, it is either expensive or infeasible to perform machining or polishing in which case (electro) chemical surface treatments could be helpful in smoothening the surface of AM parts. It has been shown that a treatment improving the surface of AM metals may significantly improve their fatigue performance [50]. However, further investigation is called for to determine what the most effective types of treatments are and to integrate those post-AM processes into the production line of AM metals.

In addition to improving the microstructure and mechanical properties of AM metals, post-AM surface treatments could be used to induce new functionalities. Although such additional functionalities could potentially be applied in various industries, the biomedical applications have received the most attention to date. For example, post-AM surface treatments and coatings have been used to induce antibacterial properties [52,53,54] and to improve tissue regeneration performance [55,56] of AM porous metallic biomaterials. Such post-AM treatments are, however, at their infancy. Their potential in other industries is under-explored as well. As for the biomedical industry, post-AM treatments and coatings could potentially be used to induce multiple functionalities at the same time. However, not so many reports of such multi-functional biomaterials currently exist.

## 5. Computational Modelling

Computational models are primarily used for two purposes, as far as the AM of metallic materials is concerned. The first purpose is modelling the AM process itself (see e.g., [57]) to simulate the interaction between the energy source and the material, to understand the evolution of the microstructure, to predict the resulting residual stresses, and to estimate the ultimate mechanical properties. The second application of computational models is in design-for-AM and is discussed in the next section (Section 6).

The utility of computational models originates from their predictive power. This predictive power allows for better understanding of the relationship between the parameters of the AM process and the properties of the resulting material. In addition to helping us establish this generic understanding of process-property relationships, computational models could be used for on the fly optimization of the process parameters as well as for geometry- and process-specific optimization of the post-AM treatments.

The main challenges in building computational models of the AM process are caused by the multi-physics, multi-scale nature of the involved problem. Modelling the interactions between the energy source (laser, electron beam) and the powder, capturing the physics and mechanics of the flow of the powder as a granular matter, simulating the heat transfer between the various types of involved materials, determining the shape and temperature profiles of the melt pool, relating the local thermal history of the melt pool to the solidification process and thermophysical changes in the microstructure of the powder material, predicting the spatial distribution of the residual stresses, describing the stochastic processes driving the formation of manufacturing irregularities, and modelling the relationship between the microstructure and the mechanical properties are all aspects that need to be taken into account when developing computational models of the AM process. Most currently available computational models capture only some of the above-mentioned effects. It is therefore essential to develop integrated models that take all of the physical phenomena determining the quality of the final AM metallic parts into account at different scales. This line of research is very challenging and has so far made only limited progress. More contributions from the groups possessing experience in computational material science, computational mechanics, physics of granular matter, and multi-scale computational modelling are needed to develop the type of computational models that could adequately address the complexity of the problem at hand.

## 6. Design-for-AM

The batch-size-indifference and complexity-for-free features of AM materials respectively enable realisation of custom-made parts and designer materials [58]. Custom-made parts are particularly interesting in the areas where one size does not fit all applications such as biomedical implants where the geometry and size of the implant should match the anatomy of the patient [59,60,61]. Since most orthopaedic implants are metallic, the design of custom-made orthopaedic implants is particularly relevant for AM metallic materials. Given the high level of individual variations in the shape of the bones [62], the patient-specific design process usually starts from medical images that need to be segmented first and be used for building 3D models of bones that will then be utilized to design the implants. There is also often a need for iterations on the design in consultation with the surgeon. This time-consuming and laborious process tremendously increases the cost of patient-specific implants. Therefore, one of the major areas that requires urgent attention is automating the design process of patient-specific implants. The same type of methodology is likely to be useful for all kinds of other applications where custom-made parts need to be designed.

AM could also be used for development of the so-called designer materials with novel properties and, thus, functionalities that originate from their complex topological design at the nano-/micro-scale [63,64]. This kind of materials are often referred to as metamaterials and may strive to achieve novel mechanical [65,66,67,68,69], acoustic [70,71,72,73], or biomedical [74,75] properties. The topological design of such metamaterials is in many cases based on various types of lattice structures. The central research question when designing metamaterials is establishing topology-property relationships that could be used to predict the type of the topological design required for achieving a specific range of physical or mechanical properties. Computational models including those based on finite element models [76,77,78] and topology optimization algorithms [79,80,81,82] as well as analytical models [83,84,85] could be very useful for establishing such topology-property relationships. However, this kind of computational models often only exist for the simplest geometries and simplest types of (mechanical) properties. For example, there are only a few computational models [86,87] available that could be used to predict the fatigue behaviour of AM metamaterials such as meta-biomaterials and mechanical metamaterials. Development of predictive models that could relate the topological design of AM metamaterials to their properties particularly the properties that are less straightforward to predict (e.g., fatigue behaviour and mass transport properties) should receive more attention in future research projects.

## 7. Conclusions

Some of the frontiers of research in AM metallic materials were briefly reviewed in this editorial to identify the most important research questions that are worthy of further investigation by researchers. The relevant literature was also cited to enable the readers to more easily find the required background information and more extensive explanation of the research questions. I hope this editorial encourages further research in this exciting area of scientific inquiry and that the time and energy of the researchers are spent answering the questions that require the most attention.

## References

[1] Zadpoor A.A., Malda J. (2017). Additive manufacturing of biomaterials, tissues, and organs. Ann. Biomed. Eng..

[2] Huang R., Riddle M., Graziano D., Warren J., Das S., Nimbalkar S., Cresko J., Masanet E. (2016). Energy and emissions saving potential of additive manufacturing: The case of lightweight aircraft components. J. Clean. Prod..

[3] Murr L.E. (2016). Frontiers of 3D printing/additive manufacturing: From human organs to aircraft fabrication. J. Mater. Sci. Technol..

[4] Uriondo A., Esperon-Miguez M., Perinpanayagam S. (2015). The present and future of additive manufacturing in the aerospace sector: A review of important aspects. Proc. Inst. Mech. Eng. Part G J. Aerosp. Eng..

[5] Fafenrot S., Grimmelsmann N., Wortmann M., Ehrmann A. (2017). Three-dimensional (3D) printing of polymer-metal hybrid materials by fused deposition modeling. Materials.

[6] Attar H., Calin M., Zhang L., Scudino S., Eckert J. (2014). Manufacture by selective laser melting and mechanical behavior of commercially pure titanium. Mater. Sci. Eng. A.

[7] Gu D., Hagedorn Y.-C., Meiners W., Meng G., Batista R.J.S., Wissenbach K., Poprawe R. (2012). Densification behavior, microstructure evolution, and wear performance of selective laser melting processed commercially pure titanium. Acta Mater..

[8] Santos E., Osakada K., Shiomi M., Kitamura Y., Abe F. (2004). Microstructure and mechanical properties of pure titanium models fabricated by selective laser melting. Proc. Inst. Mech. Eng. Part C J. Mech. Eng. Sci..

[9] Wauthle R., Ahmadi S.M., Yavari S.A., Mulier M., Zadpoor A.A., Weinans H., Van Humbeeck J., Kruth J.-P., Schrooten J. (2015). Revival of pure titanium for dynamically loaded porous implants using additive manufacturing. Mater. Sci. Eng. C.

[10] Facchini L., Magalini E., Robotti P., Molinari A., Höges S., Wissenbach K. (2010). Ductility of a Ti-6Al-4V alloy produced by selective laser melting of prealloyed powders. Rapid Prototyp. J..

[11] Vilaro T., Colin C., Bartout J.-D. (2011). As-fabricated and heat-treated microstructures of the Ti-6Al-4V alloy processed by selective laser melting. Metall. Mater. Trans. A.

[12] Xu W., Brandt M., Sun S., Elambasseril J., Liu Q., Latham K., Xia K., Qian M. (2015). Additive manufacturing of strong and ductile ti–6al–4v by selective laser melting via in situ martensite decomposition. Acta Mater..

[13] Zhao X., Li S., Zhang M., Liu Y., Sercombe T.B., Wang S., Hao Y., Yang R., Murr L.E. (2016). Comparison of the microstructures and mechanical properties of Ti–6Al–4V fabricated by selective laser melting and electron beam melting. Mater. Des..

[14] Schulze C., Weinmann M., Schweigel C., Keßler O., Bader R. (2018). Mechanical properties of a newly additive manufactured implant material based on ti-42Nb. Materials.

[15] Badrossamay M., Childs T. (2007). Further studies in selective laser melting of stainless and tool steel powders. Int. J. Mach. Tools Manuf..

[16] Casalino G., Campanelli S., Contuzzi N., Ludovico A. (2015). Experimental investigation and statistical optimisation of the selective laser melting process of a maraging steel. Opt. Laser Technol..

[17] Li R., Shi Y., Wang Z., Wang L., Liu J., Jiang W. (2010). Densification behavior of gas and water atomized 316l stainless steel powder during selective laser melting. Appl. Surf. Sci..

[18] Yan C., Hao L., Hussein A., Young P., Raymont D. (2014). Advanced lightweight 316l stainless steel cellular lattice structures fabricated via selective laser melting. Mater. Des..

[19] Demir A.G., Previtali B. (2017). Additive manufacturing of cardiovascular cocr stents by selective laser melting. Mater. Des..

[20] Hedberg Y.S., Qian B., Shen Z., Virtanen S., Wallinder I.O. (2014). In vitro biocompatibility of cocrmo dental alloys fabricated by selective laser melting. Dent. Mater..

[21] Schwindling F.S., Seubert M., Rues S., Koke U., Schmitter M., Stober T. (2015). Two-body wear of cocr fabricated by selective laser melting compared with different dental alloys. Tribol. Lett..

[22] Zhou X., Li K., Zhang D., Liu X., Ma J., Liu W., Shen Z. (2015). Textures formed in a cocrmo alloy by selective laser melting. J. Alloys Compd..

[23] Bormann T., Schumacher R., Müller B., Mertmann M., de Wild M. (2012). Tailoring selective laser melting process parameters for niti implants. J. Mater. Eng. Perform..

[24] Dadbakhsh S., Vrancken B., Kruth J.-P., Luyten J., Van Humbeeck J. (2016). Texture and anisotropy in selective laser melting of niti alloy. Mater. Sci. Eng. A.

[25] Gorgin Karaji Z., Speirs M., Dadbakhsh S., Kruth J.-P., Weinans H., Zadpoor A., Amin Yavari S. (2017). Additively manufactured and surface biofunctionalized porous nitinol. ACS Appl. Mater. Interfaces.

[26] Habijan T., Haberland C., Meier H., Frenzel J., Wittsiepe J., Wuwer C., Greulich C., Schildhauer T., Köller M. (2013). The biocompatibility of dense and porous nickel–titanium produced by selective laser melting. Mater. Sci. Eng. C.

[27] Saedi S., Turabi A.S., Andani M.T., Haberland C., Karaca H., Elahinia M. (2016). The influence of heat treatment on the thermomechanical response of ni-rich niti alloys manufactured by selective laser melting. J. Alloys Compd..

[28] Amato K., Gaytan S., Murr L., Martinez E., Shindo P., Hernandez J., Collins S., Medina F. (2012). Microstructures and mechanical behavior of inconel 718 fabricated by selective laser melting. Acta Mater..

[29] Jia Q., Gu D. (2014). Selective laser melting additive manufacturing of inconel 718 superalloy parts: Densification, microstructure and properties. J. Alloys Compd..

[30] Trosch T., Strößner J., Völkl R., Glatzel U. (2016). Microstructure and mechanical properties of selective laser melted inconel 718 compared to forging and casting. Mater. Lett..

[31] Wang Z., Guan K., Gao M., Li X., Chen X., Zeng X. (2012). The microstructure and mechanical properties of deposited-IN718 by selective laser melting. J. Alloys Compd..

[32] Li Y., Zhou J., Pavanram P., Leeflang M., Fockaert L., Pouran B., Tümer N., Schröder K.-U., Mol J., Weinans H. (2018). Additively manufactured biodegradable porous magnesium. Acta Biomater..

[33] Ng C., Savalani M., Lau M., Man H. (2011). Microstructure and mechanical properties of selective laser melted magnesium. Appl. Surf. Sci..

[34] Grasso M., Demir A., Previtali B., Colosimo B. (2018). In situ monitoring of selective laser melting of zinc powder via infrared imaging of the process plume. Robot. Comput.-Integr. Manuf..

[35] Kimura T., Nakamoto T. (2016). Microstructures and mechanical properties of A356 (AlSi7Mg0.3) aluminum alloy fabricated by selective laser melting. Mater. Des..

[36] Li Y., Gu D. (2014). Parametric analysis of thermal behavior during selective laser melting additive manufacturing of aluminum alloy powder. Mater. Des..

[37] Louvis E., Fox P., Sutcliffe C.J. (2011). Selective laser melting of aluminium components. J. Mater. Process. Technol..

[38] Mertens R., Clijsters S., Kempen K., Kruth J.-P. (2014). Optimization of scan strategies in selective laser melting of aluminum parts with downfacing areas. J. Manuf. Sci. Eng..

[39] Martin J.H., Yahata B.D., Hundley J.M., Mayer J.A., Schaedler T.A., Pollock T.M. (2017). 3D printing of high-strength aluminium alloys. Nature.

[40] Krakhmalev P., Yadroitsev I., Yadroitsava I., de Smidt O. (2017). Functionalization of biomedical Ti6Al4V via in situ alloying by cu during laser powder bed fusion manufacturing. Materials.

[41] Baitimerov R., Lykov P., Zherebtsov D., Radionova L., Shultc A., Prashanth K.G. (2018). Influence of powder characteristics on processability of AlSi12 alloy fabricated by selective laser melting. Materials.

[42] Hitzler L., Hirsch J., Heine B., Merkel M., Hall W., Öchsner A. (2017). On the anisotropic mechanical properties of selective laser-melted stainless steel. Materials.

[43] Wauthle R., Vrancken B., Beynaerts B., Jorissen K., Schrooten J., Kruth J.-P., Van Humbeeck J. (2015). Effects of build orientation and heat treatment on the microstructure and mechanical properties of selective laser melted Ti6Al4V lattice structures. Addit. Manuf..

[44] Wang P., Sin W.J., Nai M.L.S., Wei J. (2017). Effects of processing parameters on surface roughness of additive manufactured Ti-6Al-4V via electron beam melting. Materials.

[45] Kasperovich G., Hausmann J. (2015). Improvement of fatigue resistance and ductility of tial6v4 processed by selective laser melting. J. Mater. Process. Technol..

[46] Sercombe T., Jones N., Day R., Kop A. (2008). Heat treatment of ti-6al-7Nb components produced by selective laser melting. Rapid Prototyp. J..

[47] AlMangour B., Grzesiak D., Yang J.-M. (2017). Selective laser melting of TiB2/316L stainless steel composites: The roles of powder preparation and hot isostatic pressing post-treatment. Powder Technol..

[48] AlMangour B., Grzesiak D., Yang J.-M. (2017). Selective laser melting of TiB2/H13 steel nanocomposites: Influence of hot isostatic pressing post-treatment. J. Mater. Process. Technol..

[49] Leuders S., Thöne M., Riemer A., Niendorf T., Tröster T., Richard H., Maier H. (2013). On the mechanical behaviour of titanium alloy tial6v4 manufactured by selective laser melting: Fatigue resistance and crack growth performance. Int. J. Fatigue.

[50] Cutolo A., Neirinck B., Lietaert K., de Formanoir C., Van Hooreweder B. (2018). Influence of layer thickness and post-process treatments on the fatigue properties of CoCr scaffolds produced by laser powder bed fusion. Addit. Manuf..

[51] Dallago M., Fontanari V., Torresani E., Leoni M., Pederzolli C., Potrich C., Benedetti M. (2018). Fatigue and biological properties of Ti-6Al-4V eli cellular structures with variously arranged cubic cells made by selective laser melting. J. Mech. Behav. Biomed. Mater..

[52] Amin Yavari S., Loozen L., Paganelli F.L., Bakhshandeh S., Lietaert K., Groot J.A., Fluit A.C., Boel C., Alblas J., Vogely H.C. (2016). Antibacterial behavior of additively manufactured porous titanium with nanotubular surfaces releasing silver ions. ACS Appl. Mater. Interfaces.

[53] Bakhshandeh S., Gorgin Karaji Z., Lietaert K., Fluit A.C., Boel C.E., Vogely H.C., Vermonden T., Hennink W.E., Weinans H., Zadpoor A.A. (2017). Simultaneous delivery of multiple antibacterial agents from additively manufactured porous biomaterials to fully eradicate planktonic and adherent staphylococcus aureus. ACS Appl. Mater. Interfaces.

[54] Van Hengel I.A., Riool M., Fratila-Apachitei L.E., Witte-Bouma J., Farrell E., Zadpoor A.A., Zaat S.A., Apachitei I. (2017). Selective laser melting porous metallic implants with immobilized silver nanoparticles kill and prevent biofilm formation by methicillin-resistant staphylococcus aureus. Biomaterials.

[55] Nune K., Misra R., Gai X., Li S., Hao Y. (2018). Surface nanotopography-induced favorable modulation of bioactivity and osteoconductive potential of anodized 3D printed Ti-6Al-4V alloy mesh structure. Biomater. Appl..

[56] Xu J.-Y., Chen X.-S., Zhang C.-Y., Liu Y., Wang J., Deng F.-L. (2016). Improved bioactivity of selective laser melting titanium: Surface modification with micro-/nano-textured hierarchical topography and bone regeneration performance evaluation. Mater. Sci. Eng. C.

[57] Rausch A.M., Küng V.E., Pobel C., Markl M., Körner C. (2017). Predictive simulation of process windows for powder bed fusion additive manufacturing: Influence of the powder bulk density. Materials.

[58] Zadpoor A.A. (2017). Design for additive bio-manufacturing: From patient-specific medical devices to rationally designed meta-biomaterials. Int. J. Mol. Sci..

[59] Dérand P., Rännar L.-E., Hirsch J.-M. (2012). Imaging, virtual planning, design, and production of patient-specific implants and clinical validation in craniomaxillofacial surgery. Craniomaxillofac. Trauma Reconstr..

[60] Jardini A.L., Larosa M.A., Maciel Filho R., de Carvalho Zavaglia C.A., Bernardes L.F., Lambert C.S., Calderoni D.R., Kharmandayan P. (2014). Cranial reconstruction: 3D biomodel and custom-built implant created using additive manufacturing. J. Cranio-Maxillofac. Surg..

[61] Salmi M., Tuomi J., Paloheimo K.-S., Björkstrand R., Paloheimo M., Salo J., Kontio R., Mesimäki K., Mäkitie A.A. (2012). Patient-specific reconstruction with 3D modeling and dmls additive manufacturing. Rapid Prototyp. J..

[62] Sarkalkan N., Weinans H., Zadpoor A.A. (2014). Statistical shape and appearance models of bones. Bone.

[63] Hedayati R., Leeflang A., Zadpoor A. (2017). Additively manufactured metallic pentamode meta-materials. Appl. Phys. Lett..

[64] Lee J.H., Singer J.P., Thomas E.L. (2012). Micro-/nanostructured mechanical metamaterials. Adv. Mater..

[65] Berger J., Wadley H., McMeeking R. (2017). Mechanical metamaterials at the theoretical limit of isotropic elastic stiffness. Nature.

[66] Hewage T.A., Alderson K.L., Alderson A., Scarpa F. (2016). Double-negative mechanical metamaterials displaying simultaneous negative stiffness and negative poisson’s ratio properties. Adv. Mater..

[67] Kolken H.M., Janbaz S., Leeflang S.M., Lietaert K., Weinans H.H., Zadpoor A.A. (2018). Rationally designed meta-implants: A combination of auxetic and conventional meta-biomaterials. Mater. Horiz..

[68] Wang Q., Jackson J.A., Ge Q., Hopkins J.B., Spadaccini C.M., Fang N.X. (2016). Lightweight mechanical metamaterials with tunable negative thermal expansion. Phys. Rev. Lett..

[69] Zheng X., Lee H., Weisgraber T.H., Shusteff M., DeOtte J., Duoss E.B., Kuntz J.D., Biener M.M., Ge Q., Jackson J.A. (2014). Ultralight, ultrastiff mechanical metamaterials. Science.

[70] Chen H., Chan C. (2007). Acoustic cloaking in three dimensions using acoustic metamaterials. Appl. Phys. Lett..

[71] Cummer S.A., Christensen J., Alù A. (2016). Controlling sound with acoustic metamaterials. Nat. Rev. Mater..

[72] Mei J., Ma G., Yang M., Yang Z., Wen W., Sheng P. (2012). Dark acoustic metamaterials as super absorbers for low-frequency sound. Nat. Commun..

[73] Zigoneanu L., Popa B.-I., Cummer S.A. (2014). Three-dimensional broadband omnidirectional acoustic ground cloak. Nat. Mater..

[74] Ahmadi S., Hedayati R., Li Y., Lietaert K., Tümer N., Fatemi A., Rans C., Pouran B., Weinans H., Zadpoor A. (2018). Fatigue performance of additively manufactured meta-biomaterials: The effects of topology and material type. Acta Biomater..

[75] Hedayati R., Ahmadi S., Lietaert K., Pouran B., Li Y., Weinans H., Rans C., Zadpoor A. (2018). Isolated and modulated effects of topology and material type on the mechanical properties of additively manufactured porous biomaterials. J. Mech. Behav. Biomed. Mater..

[76] Cho H.-H., Cho Y., Han H.N. (2015). Finite element analysis for mechanical response of ti foams with regular structure obtained by selective laser melting. Acta Mater..

[77] Dong G., Tang Y., Zhao Y.F. (2017). A survey of modeling of lattice structures fabricated by additive manufacturing. J. Mech. Des..

[78] Kadkhodapour J., Montazerian H., Darabi A.C., Anaraki A., Ahmadi S., Zadpoor A., Schmauder S. (2015). Failure mechanisms of additively manufactured porous biomaterials: Effects of porosity and type of unit cell. J. Mech. Behav. Biomed. Mater..

[79] Lin C.Y., Wirtz T., LaMarca F., Hollister S.J. (2007). Structural and mechanical evaluations of a topology optimized titanium interbody fusion cage fabricated by selective laser melting process. J. Biomed. Mater. Res. Part A.

[80] Wang Y., Xu H., Pasini D. (2017). Multiscale isogeometric topology optimization for lattice materials. Comput. Methods Appl. Mech. Eng..

[81] Xiao D., Yang Y., Su X., Wang D., Sun J. (2013). An integrated approach of topology optimized design and selective laser melting process for titanium implants materials. Bio-Med. Mater. Eng..

[82] Xiao Z., Yang Y., Xiao R., Bai Y., Song C., Wang D. (2018). Evaluation of topology-optimized lattice structures manufactured via selective laser melting. Mater. Des..

[83] Hedayati R., Sadighi M., Mohammadi-Aghdam M., Zadpoor A. (2016). Mechanical properties of regular porous biomaterials made from truncated cube repeating unit cells: Analytical solutions and computational models. Mater. Sci. Eng. C.

[84] Hedayati R., Sadighi M., Mohammadi-Aghdam M., Zadpoor A. (2016). Mechanics of additively manufactured porous biomaterials based on the rhombicuboctahedron unit cell. J. Mech. Behav. Biomed. Mater..

[85] Hedayati R., Sadighi M., Mohammadi-Aghdam M., Zadpoor A. (2016). Mechanical behavior of additively manufactured porous biomaterials made from truncated cuboctahedron unit cells. Int. J. Mech. Sci..

[86] Hedayati R., Hosseini-Toudeshky H., Sadighi M., Mohammadi-Aghdam M., Zadpoor A. (2016). Computational prediction of the fatigue behavior of additively manufactured porous metallic biomaterials. Int. J. Fatigue.

[87] Hedayati R., Hosseini-Toudeshky H., Sadighi M., Mohammadi-Aghdam M., Zadpoor A. (2018). Multiscale modeling of fatigue crack propagation in additively manufactured porous biomaterials. Int. J. Fatigue.

